# A Surgical Challenge Generated by Colonic Malakoplakia in Disguise as a Locally Advanced Colonic Malignancy—A Case Report

**DOI:** 10.3390/medicina59010156

**Published:** 2023-01-12

**Authors:** Cristina Șerban, Alexandra Toma, Dragoș Cristian Voicu, Constantin Popazu, Dorel Firescu, George Țocu, Raul Mihailov, Laura Rebegea

**Affiliations:** 1 Surgical Department, Faculty of Medicine and Pharmacy, “Dunărea de Jos” University of Galați, 800201 Galați, Romania; 2 Surgical Clinical Department, “Sf. Apostol Andrei” Emergency Clinical County Hospital, 800578 Galați, Romania; 3 Surgical Clinical Department, Emergency Clinical County Hospital of Braila, 810325 Braila, Romania; 4 Pharmaceutical Sciences Department, “Sf. Apostol Andrei” Emergency Clinical County Hospital, 800578 Galați, Romania; 5 Radiotherapy Department, “Sf. Apostol Andrei” Emergency Clinical County Hospital, 800578 Galați, Romania

**Keywords:** malakoplakia, colon, Michaelis-Gutmann bodies, colorectal surgery, intestinal obstruction

## Abstract

Colonic malakoplakia is an uncommon granulomatous development of cells resulting from the impaired capacity of the mononuclear cells to eliminate the phagocytosed bacteria, and in rare cases it can also affect the gastrointestinal tract. We report the case of a 78-year-old female patient that was admitted to hospital by The Emergency Department with the diagnosis of bowel obstruction, confirmed by the clinical and paraclinical investigations. We decided to surgically manage the case for suspicious symptomatic colonic neoplasm. The histological examination of the surgical specimens revealed colonic malakoplakia, characterized by the presence of the aggregated granular histiocytes and Michaelis-Gutmann bodies. Through this paper, we want to raise awareness for Malakoplakia, which remains an extremely rare disease that may affect multiple organs, and because it does not present specific symptoms or clinical manifestations, the final diagnosis remains the histopathological study. The clinical conduct should be decided after taking into consideration all the aspects of this pathology along with the benefits and risks for the patient.

## 1. Introduction

Malakoplakia was described for the first time by Michaelis and Gutmann in 1902 [1]. One year later, von Hansemann specified the term “malakoplakia”, derived from the Greek “malakos” (soft) and “plakos” (plate) [1].

This pathology is more frequently encountered in the urinary tract—about 75% of the reported literature cases. The gastrointestinal tract is the second most common site for the lesions, particularly the descending colon, sigmoid and rectum [2,3]. Malakoplakia can also be identified in various sites, such as the liver, pancreas, retroperitoneum, respiratory tract, genital organs, lymph nodes and even in the brain [4].

In our days, knowing the etymology of this rare pathology, we might state that the clinical appearance varies along the gastrointestinal tract and it can be identified as flat lesions, mucosal erosions or plaques, ulcerations or even polyps of different sizes. In some cases, patients might present tumors with characteristics of malignancy [5,6].

Colonic Malakoplakia was defined in most patients as part of a preexisting pathology like the diverticular disease or the ulcerative colitis, or in rare, isolated cases associated with a rectal or sigmoid colon adenocarcinoma. It has a non-specific clinical presentation in close relationship with the involved organ or region and it might result in symptoms such as abdominal pain, diarrhea, gastrointestinal bleeding or even bowel obstruction mimicking a colonic neoplasia [7].

In the pathogenesis of the malakoplakia are supposed to be involved the deficiency of guanosine monophosphate dehydrogenase and the deficiency of beta-glucuronidase, which modifies the microtubular and lysosomal function resulting in the incomplete elimination of bacteria from the macrophages. Current evidence indicates a connection with the activity of the macrophages.

The human *β*-glucuronidase is a type of glucuronidase, a member of the glycosidase 2 family, which catalyzes the hydrolysis of *β*-D-glucuronic acid residues from the non-translational end of mucopolysaccharides [4]. It is located in the lysosomes and it is essential for effective lysosomal bactericidal activity. In the intestine, the *β*-glucuronidase is involved in the conversion of the conjugated bilirubin to the unconjugated form for reabsorption. The human *β*-glucuronidase is homologous to the enzyme Escherichia coli *β*-galactosidase [5].

The guanosine monophosphate-dehydrogenase is a regulator of intracellular guanine nucleotides and it is therefore important in DNA and RNA synthesis. The synthesis of guanine nucleotides is essential for maintaining normal cell function and growth. It is also important for maintaining cell proliferation and immune responses. B and T lymphocytes depend on guanosine monophosphate dehydrogenase for normal activation and function [6,7].

The macroscopic aspect of the malakoplakia lesion is solid, soft, friable, yellow or brown-yellow, of different sizes and with different distribution patterns. From a histological point of view, there is a diffuse histiocytic infiltration with eosinophilic granular cytoplasm (von Hansemann cells), containing characteristic basophilic laminated cytoplasmic inclusions, called Michaelis-Gutmann bodies, which are specifically colored in PAS staining (Schiff Periodic Acid) [8,9,10].

Malakoplakia is caused by deficits in phagocytic or degradative functions of the macrophages, as a response to the infections with Gram-negative coliform bacteria (Escherichia coli, Staphylococcus Aureus, Mycobacterium Tuberculosis or Proteus mirabilis), which may lead to chronic inflammatory status, followed by accumulation of intracellular iron and calcium deposits that create mineralization (Michaelis-Gutmann bodies) [10,11].

The clinicians must be aware of the presence of this unusual pathology along the gastrointestinal tract. When the lesion can be identified as a polyp, nodule, presents ulcerations or is associated with lymph node involvement, malakoplakia can be very easily mistaken as a malignancy. The literature contains cases in which the patients diagnosed with malakoplakia presented various associations between colorectal adenocarcinoma, adenomas, systemic diseases or without any link with another pathology [12].

The diagnosis can be very challenging, and in the majority of the cases stated in the literature, the pathological report was unexpected. In this paper, we are illustrating a case of colonic malakoplakia that was mimicking a locally advanced colorectal neoplasm, without any association with a colorectal adenocarcinoma, an adenoma or a systemic disease, which makes it a rare occurrence.

## 2. Case Report

T.D., a 78-year-old patient admitted by emergency in the 2nd Clinical Surgery Department of “Sf. Apostol Andrei” Emergency Clinical County Hospital Galati, for diffuse abdominal pain, absence of the intestinal transit, nausea and vomiting, symptoms which have started three days before presentation to the hospital.

The laboratory examinations did not capture any pathological changes: WBC 5.560/mmc, Hb 11.8 g/dl, Ht 37.7%, PLT 252,000/mmc, Glycemia 99 mg/dl, AST 15 U/L, ALT 15 U/L, creatinine 1.17 mg/dl and urea 40 mg/dl.

The abdominal radiography revealed hydroaerial levels in the right side of the abdomen and hypogastrium.

The abdominal ultrasound showed:

- left liver lobe = 58 mm,
- right lobe of the liver = 144 mm, with slightly increased echogenicity and microgranular structure,
- PV (portal vein) = 12 mm,
- CBP (main biliary duct) = 5 mm,
- non-dilated CBIH (intrahepatic biliary duct),
- folded gallbladder in lower 1/3, with micro calculus of 5–6 mm,
- homogeneous pancreas,
- homogeneous spleen with 81 mm long axis,
- RK (right kidney) of normal size, IP retained, calculi of 5–6 mm, no pyelocaliceal dilations,
- LK (left kidney) of normal size, IP retained, calculi of 5–6 mm, no pyelocaliceal dilations,
- the urinary bladder half-full, without changes,
- marked aerocolia on the colic frame, without free intraperitoneal fluid.

We also performed an abdominal and pelvic computed tomography enhanced with contrast agent, which described a marked distension with hydroaerial levels and parietal aerial inclusions (pneumatosis) involving the intestinal loops, cecum and ascending colon, up to the hepatic angle, where a 10 mm thickened wall area was highlighted.

Given the symptoms of the patient and the paraclinical findings and established clinical diagnosis of bowel obstruction, we decided to manage this case by performing an emergency surgery.

During surgery, after close inspection of the whole abdominal cavity, the team identified a stenosing mass located in the hepatic angle of the ascending colon and subsequent bowel obstruction. We decided that a right hemicolectomy with ileotransversoanastomosis was the best surgical approach for our patient and we performed it.

**Figure 1:**
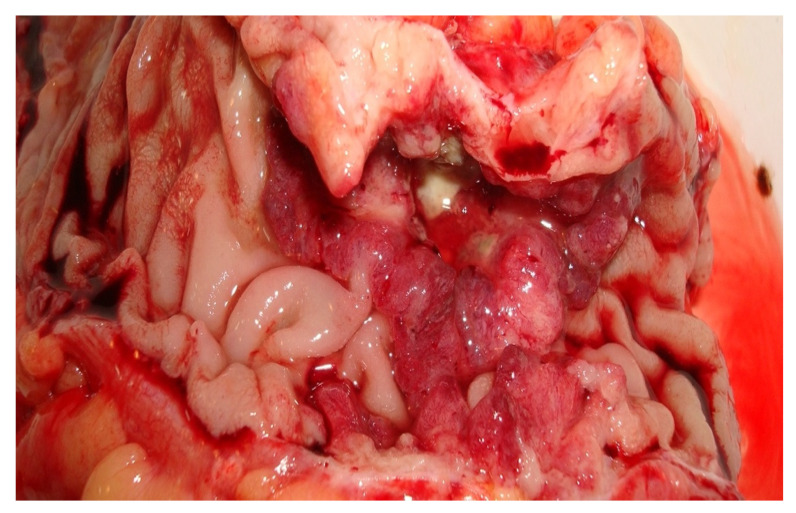
The appearance of the resected portion (2nd Surgery Clinic, patient T.D.).

Figure 1 illustrates the appearance of the portion of the bowel that was surgically resected and was set to the anatomopathological laboratory for histological diagnosis.

The postoperative evolution of the patient was favorable and uneventful, with the improvement of the general state, decreasing need for painkillers, active mobilization and good digestive tolerance.

The patient was discharged after 14 days, and was aware and cooperative and in good general state.

## 3. Pathologic Findings

**Figure 2:**
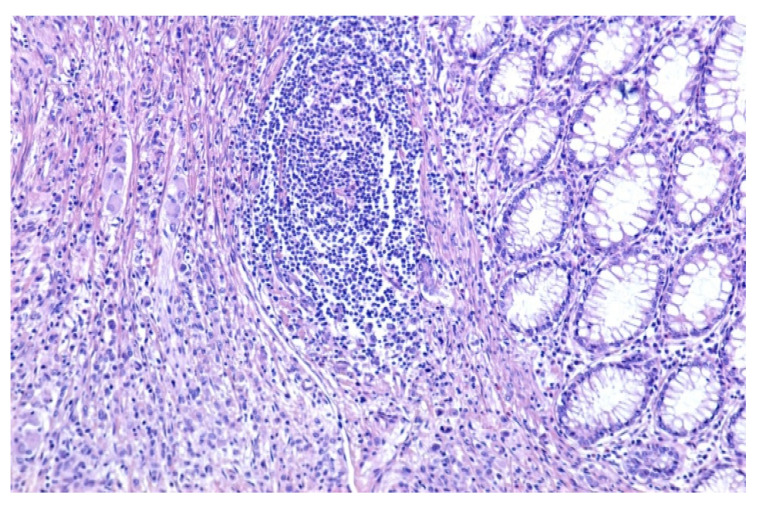
Colonic mucosa, which presents at the lamina propria level abundant mononuclear inflammatory infiltrate, predominantly macrophagic (HE, ×100).

**Figure 3:**
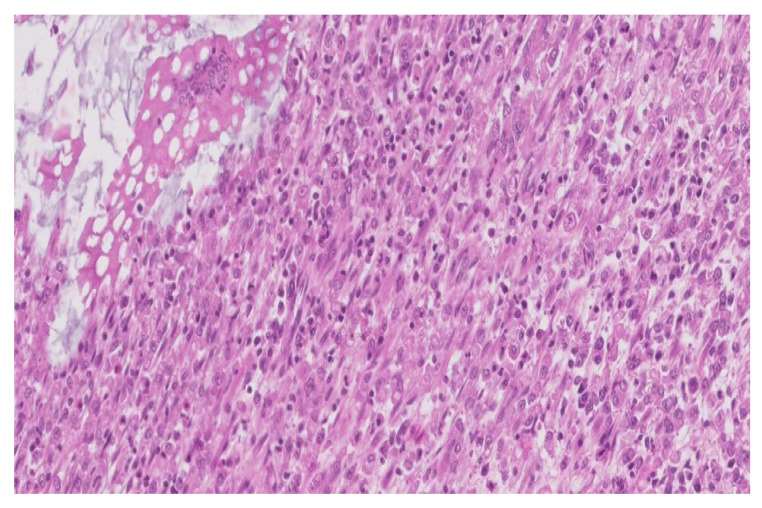
Dense macrophage infiltrate with rare dispersed lymphocytes (HE, ×200).

The histopathological diagnostic of the postoperator specimen (4394) was colonic malakoplakia as illustrated in Figure 2 and Figure 3.

The microscopic examination revealed:

- a significant architectural remodeling of the colonic mucosa and submucosa with dense cellular inflammatory infiltrate,
- the dense cellular inflammatory infiltrate-composed of epithelioid macrophages with wide eosinophilic cytoplasm, variable-sized nuclei, with occasional nucleoli,
- dispersed syncytia with the appearance of multinucleated giant cells, with chaotically disposed nuclei and
- amorphous cytoplasmatic inclusions of cellular detritus type, with sporadic intercalated lymphocytes.

Neither areas of necrosis, nor viable microorganisms were detected.

An area of ulcerated mucosa was identified, with reactive changes at the level of the remaining marginal epithelium.

Additionally, no invasive dysplastic or neoplastic stigmata were identified.

Furthermore, the anatomopathological department recommended immunohistochemical tests for a precise and definitive diagnosis of our patient.

**Figure 4:**
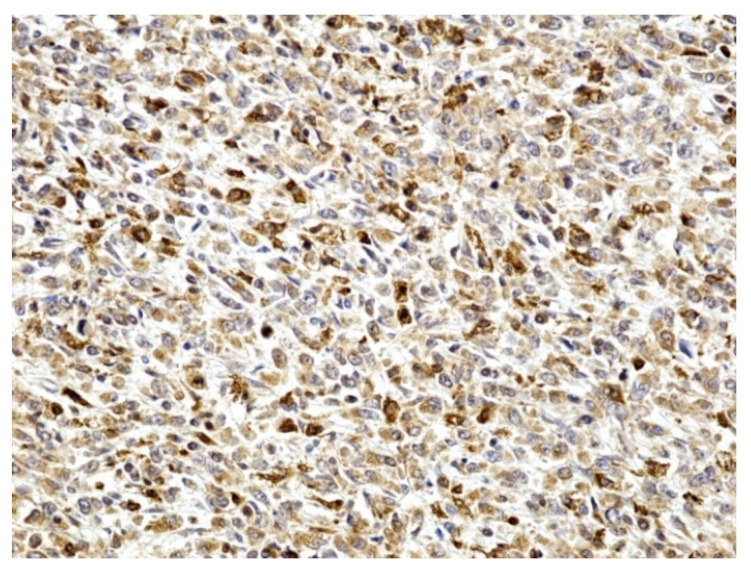
Positive CD68 in the macrophage infiltrate (×200).

The result of the immunohistochemical tests confirmed that the inflammatory infiltrate was predominantly comprised of:

- macrophages (positive CD68)-illustrated in the Figure 4
- with rare dispersed T lymphocytes (positive CD3),
- rare B lymphocytes (alpha positive CD20, CD79) especially in the lamina propria,
- rare plasmacytes (alpha positive CD79),
- as well as rare mast cells (positive tryptase).

**Figure 5:**
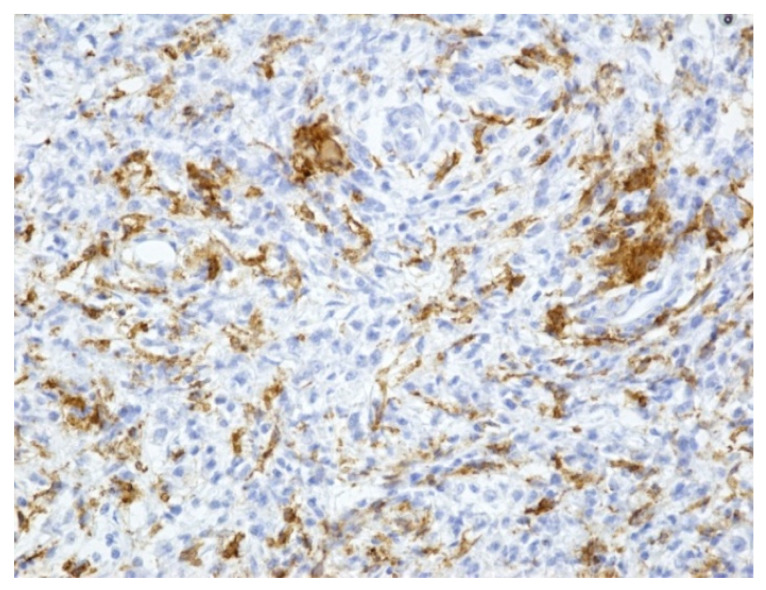
Positive CD204 in most macrophages (×200).

Additionally, most macrophages were positive for CD204 (MSR1), a receptor that plays a role in phagocytosis (Figure 5).

Alpha-SMA was positive in the smooth muscle cells, from the level of the muscularis mucosae and the own muscle, being negative in the inflammatory cells.

## 4. Discussions

Malakoplakia represents a rare type of granulomatous inflammatory pathology that can affect multiple sites in the human body. The most frequent localization is the genitourinary tract, the digestive system, but also the nervous system, the respiratory organs and even the bones.

It is most frequently encountered in patients diagnosed with chronic pathologies, like the ones illustrated in Table 1.

The most distinctive histological feature for the diagnostic of malakoplakia is represented by the Michaelis-Gutmann bodies, which are concentric, basophilic, round or oval lamellae, with a diameter of 5 to 10 µm, included in the cytoplasm of the histocytes, known as von Hansemann cells. These inclusions are generally positive PAS and von Kossa, being composed of a glycolipid matrix, probably of a specific microorganism and are covered with a calcium and iron layer. The foamy histiocytes are mainly dispersed in the lamina propria of the mucosa, next to rare lymphocytes and occasionally to multinucleated giant cells. Therefore, the precise diagnosis of this pathology can only be stated after microscopic examination, in the usual hematoxylin eosin staining, as well as by using the mentioned special staining, or by performing immunohistochemical tests [11].

The literature data is illustrated by case reports and some small case series that bring light regarding this rare disease, its forms of presentation, the features of the patients diagnosed with it, their symptoms and the type of medical conduct that could be applied.

We performed a PubMed search of other reported cases of digestive tract malakoplakia, especially the ones involving the colon, in order to illustrate the features of this rare pathology. We excluded the pediatric cases and the articles that could not be processed (no text available). The results are shown in Table 2.

As summarized in Table 2, the current knowledge about this rare and uncommon pathology is mainly based on case reports and small series due to the rareness of Malakoplakia. About 90% of the literature cases involve the colon as the main site for pathologic degeneration of the structures, with a median age presentation of about 64 years old and a higher incidence between female patients-63%. We included in the table only the cases that involved colonic malakoplakia, regardless of the other pathologic conditions of the patient, without any association between malakoplakia and adenocarcinoma or other digestive tract malignancy, because just like the case we reported, these are very rare cases and most of them are incidental findings.

The etiology of malakoplakia is still in debate, but the specialty literature mentions possible pathogenic status like infections which involve Gram-negative pathogens, in particular E. coli and an abnormal response of the immune system, most probably generated by inefficient lysosomal functions of the macrophages [9,10].

The clinical manifestations of the colonic malakoplakia depend on the extent of the colonic lesions and the coexisting pathology of the patient. They are nonspecific and include:

- rectal bleeding,
- diarrhea,
- abdominal pain in different grades,
- fever,
- weight loss in the recent period before presenting to the hospital,
- night sweats and
- intestinal tract obstruction.

Three types of pathologic findings are described by endoscopic examination:

1. Sessile or polypoid masses isolated in the rectosigmoid colon; the lumen may be narrowed which may suggest malignant stenosis. The inflammatory process may be transmural, similar to the Crohn’s disease.
2. Diffuse involvement of the entire colon either with serpiginous polypoid lesions or with diffuse ulcerations; variant often identified in patients with compromised immunity, including the patients with renal transplant, immunosuppressive drug therapy and hereditary immunodeficiency.
3. A single mass or in association with colonic neoplasms or adenomatous polyps. The incidence of colon malignancy associated with intestinal malakoplakia can reach up to 35% [2,11].

Colonic malakoplakia can mimic other pathologies, both macroscopically and microscopically and it is very important that before deciding the right management for the patient, the medical team must also take into consideration the differential diagnosis. The differential diagnosis after macroscopic examination may include:

- malignant tumors,
- miliary tuberculosis and
- Crohn’s disease.
- Microscopically, the malakoplakia may be similar to:
- the Whipple disease,
- ceroid-like colonic histiocytosis,
- Wolman disease,
- Chediak-Higashi syndrome,
- sarcoidosis, or
- other granulomatous diseases [12].

The therapeutic management of the Malakoplakia varies from the medical therapy that includes antibiotics and surgical approach with different surgical techniques associated in the relationship with the patient’s needs. At this moment, there is no therapy considered the gold standard for this rare disease. Therapeutic alternatives are:

- antibiotic therapy using agents that interact directly with the macrophages, like the quinolones or trimethoprim-sulfamethoxazole
- diminution or even suspension of the doses prescribed for the immunosuppressive therapy
- a good control of the associated immunosuppressive pathology
- endoscopic resections for the cases with localized lesions
- surgical resection for the cases that present multiple organ infiltration and or suspected malignancy [12,13,14,15,16,17,18,19,20,21,22,23,24,25,26,27,28,29,30,31,32,33,34,35,36,37,38,39,40].

## 5. Conclusions

In conclusion, Malakoplakia is an uncommon and rare granulomatous disease that can compromise any organ or structure in the human body. It is mostly encountered in the urinary tract and the gastrointestinal system being the second most frequent site involved, particularly the descending colon, sigmoid and rectum.

The Michaelis-Gutmann bodies are pathognomonic for this disease, but they may be missing in the third stage of the Malakoplakia, when there is a progressive fibrous tissue, which may lead to bowel obstruction. 

The cases described in the literature state that this pathology affects the patients that have chronic diseases and immunosuppressive conditions. Due to the fact that the symptoms are non-specific, malakoplakia is generally identified postoperatively, from the histopathological analysis of the surgical specimen, just like the case that we illustrated.

A good knowledge of this uncommon pathology may lead, in the future, to a preoperative diagnosis, less invasive investigations and may also avoid unnecessary surgical management.

## Figures and Tables

**Table 1:** Conditions associated with Malakoplakia.

organ transplantation
neoplasms
ulcerative colitis
malnutrition
tuberculosis
allergic conditions
sarcoidosis
poorly controlled diabetes
cytotoxic chemotherapy
acquired immunodeficiency syndrome (AIDS)
steroid use
alcohol abuse

**Table 2:** Summary of features of colonic malakoplakia in the specialty literature [12,13,14,15,16,17,18,19,20,21,22,23,24,25,26,27,28,29,30,31,32,33,34,35,36,37,38,39,40].

Reference Article Number	Patient Gender	Patient Age	Clinical Presentation	Additional Findings	Location of the Lesion/s	Therapeutic Conduct	Evolution of the Patient
1	female	72	abdominal pain, vaginal bleeding	cadaveric renal transplant for chronic renal failure (10 months previously)	mesocolon—one adjacent to the descending colon and another involving the sigmoid colon	resection of the sigmoid lesion and a cutaneous fistula for the descending colon lesion	9 months of antibiotic therapy relapse of the masses with the persistence of the lesions
2	male	33	painless hematochezia	warfarin therapy for lower-extremity deep venous thrombosis and a complex neurosurgical history, chemotherapy and radiation	nonobstructing circumferential mass in the rectum	biopsies and glucocorticoid therapy, followed by bowel resection	exitus by severe sepsis due to a urinary tract infection
3	female	61	lower respiratory tract infection	diabetes mellitus, hypertension and hyperlipidemia	multiple sessile and inflammatory colonic polyps with ulcerative edges	biopsies and antibiotic therapy	persistent cluster of polyps at rectosigmoid junction with no evidence of malakoplakia
4	male	59	asymptomatic	anemia	sessile polyp in the sigmoid colon and a second one within the ascending colona mass involving the pancreatic head	resection of the colonic polyps, antibiotic therapy, Whipple procedure	improvement after surgery recurrent ascites 3 months after surgery
5	26 cases range from 24 to 83 years old	16 women 10 men	abdominal pain, diarrhea, appendicitis	—	colorectum appendix stomach	biopsies and resections	favorable evolution with no additional instances of malakoplakia
6	male	37	chronic diarrhea	edematous-ascitic syndrome and bilateral pleurisy	tumoral stenosis of the sigmoid-colic junction	wide left colectomy	improvement after surgery
7	female	55	massive hemorrhage of the rectum	—	duodenum, colon and lymph nodes in the mesentery	laparotomy and proctocolectomy	—
8	male	66	abdominal pain and chronic intermittent diarrhea	compensated alcoholic liver disease, hypertension, emphysema, chronic obstructive pulmonary disease and irritable bowel syndrome	multiple polyps in the ascending, descending and sigmoid colon cecal mass	biopsies laparoscopic right hemicolectomy	—
9	female	38	lower gastrointestinal hemorrhage	diabetes	splenic flexure mass involving the spleen and the left kidney	biopsies antibiotic therapy laparoscopic en bloc colectomy and partial nephrectomy	pancreatic fistula postoperatively, successfully treated with percutaneous drainage and antibiotics
10	male	68	abdominal pain	—	incarcerated ventral hernia and sigmoid-colon rupture	Hartmann’s procedure, antibiotic therapy associated with cholinergic agonists (bethanechol and ascorbic acid)	reversal of the Hartmann’s procedure 4 months after the initial surgery
11	male	65	abdominal pain and unintentional weight loss	—	lesion of the transverse colon	antibiotic therapy	—
12	female	19	low abdominal pain and multiple loose stools with blood for the last 3 months	—	multiple polyps throughout the colon, particularly in the sigmoid colon	antibiotic therapy associated with cholinergic agonists (bethanechol and ascorbic acid) for 6 months	abnormal colonoscopy findings were remarkably improved after 6 months of follow-up, and returned to normal after the end of 12 months currently healthy
13	male	38	6 months history of diarrhea associated with fecal incontinence	cardiac transplant 4 years previously for severe heart failure due to dilated cardiomyopathy	upper gastrointestinal endoscopy was normal and the mucosa of ileum and colon was colonoscopically normal	rectal biopsies and antibiotic therapy	1 month later, the patient did not have diarrhea
14	female	62	melena	—	polypoid lesions of the cecum	antibiotic therapy	no melena detectable over a period of 6 months
15	female	—	—	30-years history of ulcerative colitis	—	proctocolectomy after failure of medical therapy	—
16	male	58	abdominal cramps and chronic diarrhea	unintentional weight loss, Sjogren syndrome and lupus nephritis	multiple polyps in the rectum	biopsies and antibiotic therapy	symptoms improved after 3 months of follow-up
17	male	—	persistent diarrhea and recurrent E coli bacteremia	dual stem cell and cardiac transplant recipient	areas of colonic thickening	biopsies antimicrobial therapy and reduction of immunosuppression	exitus from sepsis
18	female	58	chronic diarrhea	liver transplant recipient	patchy mucosal edema	biopsies antibiotic therapy and reduction of immunosuppression	—
19	female	51	chronic recurrent diarrhea	kidney transplant surgery and hypertension	flat elevated lesions involving the ascending colon	biopsies and corticosteroids therapy	no recurring symptoms during the 9-month follow-up
20	female	55	routine cancer surveillance	liver transplant and a second liver transplant 10 months later	diffuse wall thickening of the sigmoid colon	biopsies no therapy	no symptoms at the 1 year check-up
21	female	45	diarrhea and unintentional weight loss	variable immunodeficiency and interstitial lung disease	multiple mucosal nodules in the rectum	biopsies and antibiotic therapy	improvement of the symptoms
22	female	44	—	ulcerative colitis	colon	discontinuation of high-dose systemic steroid	gradually remissions of the symptoms
23	male	56	palpable, movable and painful tumoral mass in the right iliac fossa	paracoccidioidal osteomyelitis of the right clavicle and the 6th left rib one and two years previously	mucosal irregularities and wall stiffness in the cecum and diverticulosis of the colon	—	exitus by bilateral bronchopneumo- nia and acute renal failure
24	female	78	diarrhea and malaise	anemia and elevated inflammatory parameters	macroscopic yellowish nodular changes throughout the colon	biopsies and antibiotic therapy	remission of the pathology after 3 months of antibiotic therapy
25	female	58	—	ulcerative colitis	colonic perforation was suspected	sub-total colectomy and antibiotic therapy	gradually disappearance of malakoplakia
26	female	47	—	emaciated	the ascending and transverse portions of the colon	—	exitus by large intestinal obstruction syndrome
27	male	37	rectal bleeding	stage IV Hodgkin disease	sigmoid colon	biopsies	—
28	male	22	fever, rectal bleeding with fistula	inflammatory bowel disease	pseudo tumoral masses and multiple colorectal ulcerations	biopsies and colonic diversion associated with broad spectrum antibiotherapy	favorable outcome
29	male	75	intense abdominal pain	elevated serum creatinine, elevated glycated hemoglobin and urinary infection	parietal thickening of the descending colon, left kidney, iliopsoas muscle and retroperitoneum involvement	surgery for symptomatic colonic neoplasm—left segmental colectomy, left partial nephrectomy and retroperitoneal soft tissue resection	—
30	23 cases 11 males and 12 females	mean age 57	diagnostic screening (n = 7) diarrhea (n = 3) rule out rejection (n = 1) gastrointestinal bleeding (n = 1) abdominal pain (n = 1) follow up of Barrett’s esophagus (n = 1)	most patients were immuno-suppressed (organ transplantation n = 4, cancer n = 9, autoimmune condition n = 5 and/or medication effect	sigmoid colon, rectum (n = 10) transverse and descending colon (n = 4) stomach/gastroesophageal junction (n = 4) appendix (n = 2) cecum (n = 1) small bowel (n = 1) perianal area (n = 1)	biopsies and resections	—

## Data Availability

The datasets generated and/or analyzed during the current study are not publicly available due ethical reasons but are available from the corresponding author upon reasonable request.
